# Fragment-Based Ligand Discovery Applied to the Mycolic Acid Methyltransferase Hma (MmaA4) from *Mycobacterium tuberculosis*: A Crystallographic and Molecular Modelling Study

**DOI:** 10.3390/ph14121282

**Published:** 2021-12-08

**Authors:** Romain Galy, Stéphanie Ballereau, Yves Génisson, Lionel Mourey, Jean-Christophe Plaquevent, Laurent Maveyraud

**Affiliations:** 1 Institut de Pharmacologie et de Biologie Structurale, Université Toulouse III—Paul Sabatier, Centre National de la Recherche Scientifique, 31077 Toulouse, France; romain.galy@riseup.net (R.G.); lionel.mourey@ipbs.fr (L.M.); 2 Laboratoire de Synthèse et Physico-Chimie de Molécules d’Intérêt Biologique, Université Toulouse III—Paul Sabatier, Centre National de la Recherche Scientifique, 31062 Toulouse, France; ballereau@chimie.ups-tlse.fr (S.B.); genisson@chimie.ups-tlse.fr (Y.G.); chris.plaquevent@gmail.com (J.-C.P.)

**Keywords:** *Mycobacterium tuberculosis*, mycolic acid methyltransferases, fragment-based ligand discovery, binding energies, molecular modelling

## Abstract

The mycolic acid biosynthetic pathway represents a promising source of pharmacological targets in the fight against tuberculosis. In *Mycobacterium tuberculosis*, mycolic acids are subject to specific chemical modifications introduced by a set of eight S-adenosylmethionine dependent methyltransferases. Among these, Hma (MmaA4) is responsible for the introduction of oxygenated modifications. Crystallographic screening of a library of fragments allowed the identification of seven ligands of Hma. Two mutually exclusive binding modes were identified, depending on the conformation of residues 147–154. These residues are disordered in *apo*-Hma but fold upon binding of the S-adenosylmethionine (SAM) cofactor as well as of analogues, resulting in the formation of the short η1-helix. One of the observed conformations would be incompatible with the presence of the cofactor, suggesting that allosteric inhibitors could be designed against Hma. Chimeric compounds were designed by fusing some of the bound fragments, and the relative binding affinities of initial fragments and evolved compounds were investigated using molecular dynamics simulation and generalised Born and Poisson–Boltzmann calculations coupled to the surface area continuum solvation method. Molecular dynamics simulations were also performed on *apo*-Hma to assess the structural plasticity of the unliganded protein. Our results indicate a significant improvement in the binding properties of the designed compounds, suggesting that they could be further optimised to inhibit Hma activity.

## 1. Introduction

*Mycobacterium tuberculosis* (Mtb), the causative agent of tuberculosis (TB), remains one of the deadliest infectious agents worldwide: it claimed 1.5 million deaths in 2020, and an estimated 10 million new cases were reported [1]. This remarkable efficacy as a human pathogen relies in part on the structure of its thick, atypical, highly hydrophobic cell wall [2], which limits antibiotic penetration [3], protects Mtb from the host immune system [4,5], and provides important virulence factors [6,7]. This cell wall is formed by the mycomembrane, or mycobacterial outer membrane, which surrounds arabinogalactan and peptidoglycan [2]. The inner leaflet of the mycomembrane comprises mycolic acids (MAs) covalently bound to arabinogalactan, whereas trehalose-bound mycolic acids are found in the outer leaflet [8].

Mycolic acids, long-chain 2-alkyl, 3-hydroxy fatty acids, are an idiosyncrasy of the genus *Mycobacterium* [6], and, as such, their metabolism is a relevant target in the fight against Mtb [9,10]. Indeed, isoniazid, one of the most widely used antitubercular drugs, targets this biosynthetic pathway [11,12,13]. The biosynthesis of MAs starts with the synthesis of C_16_–C_18_ fatty acids (FAs), by the multifunctional fatty acid synthase (FAS) I enzyme, which are further elongated up to C_48_–C_62_ by the FAS-II multienzyme system, while being decorated at two distinct positions by a set of eight MA S-adenosylmethionine (SAM) dependent methyltransferases (MAMTs). The enzyme Pks13 condensates these long modified FAs, called a meromycolic chain, with a C_24_–C_26_ long FA, also generated by FAS-I [14]. The resulting decorated MAs are translocated to the periplasm by the membrane transporter MmpL3 [15].

The introduction of decorations at the distal and proximal positions on the meromycolic chain necessitates the presence of *cis* double bonds. The exact mechanism that leads to the presence of these double bonds is subject to debate [6]. These *cis* double bonds can be converted at the distal and proximal positions into cyclopropane by MmaA2 [16] and PcaA [17], respectively, into a *trans* double bond with a vicinal methyl by UmaA1 [18], or hydrated into a hydroxylated compound by MmaA4/Hma [19,20]. The resulting hydroxymycolates can be further modified to keto- and methoxy-MAs by MmaA3 [19,21,22]. The catalytic mechanisms of CmaA2, MmaA4, and MmaA1 have been studied by QM/MM steered molecular dynamics [23]. It would begin with the formation of a carbocation at the olefin site that would spontaneously convert into a methyl alcohol in the case of Hma/MmaA4 [23]. Deletion of individual genes encoding SAM-dependent MAMTs is not lethal and affects the mycomembrane structure and/or virulence of Mtb to varying extent [16,17,18,24]. On the other hand, simultaneous inactivation of all eight genes encoding MAMTs resulted in a viable but highly attenuated and hyperinflammatory Mtb [25]. Furthermore, chemical inhibition of MAMTs was found to be bactericidal [24,26]. All these results suggest that MAMTs are attractive targets in the fight against TB.

Among these, MmaA4/Hma is particularly interesting, as it has been shown that it is necessary and sufficient for the introduction of oxygenated modifications on MAs [19,20,27] and that oxygenated MAs participate in the virulence of Mtb in mice [19], modulate IL12 production in macrophages [28], and trigger the differentiation of macrophages into foamy macrophages in granulomas in vitro [29]. In continuation of our previous work on the 3D structure of Hma in the presence of SAM and of cofactor analogues [26,30], we screened a small library of fragments against Hma using X-ray crystallography. Molecular dynamics simulations were performed for the experimentally observed bound fragments to estimate their binding energies. Based on the observed structures, evolved fragments were designed and their binding energies were also estimated.

## 2. Results

### 2.1. Crystallographic Structures of Fragment-Bound Hma

**Figure 1:**
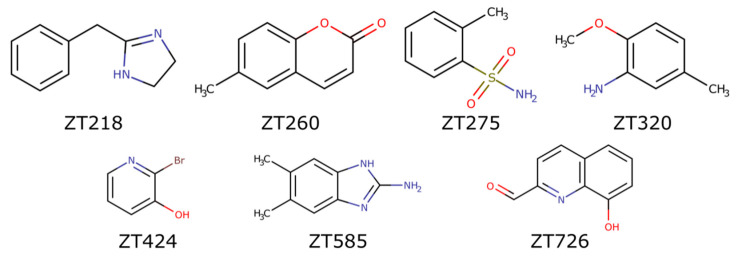
Structures of the fragments bound to Hma.

**Figure 2:**
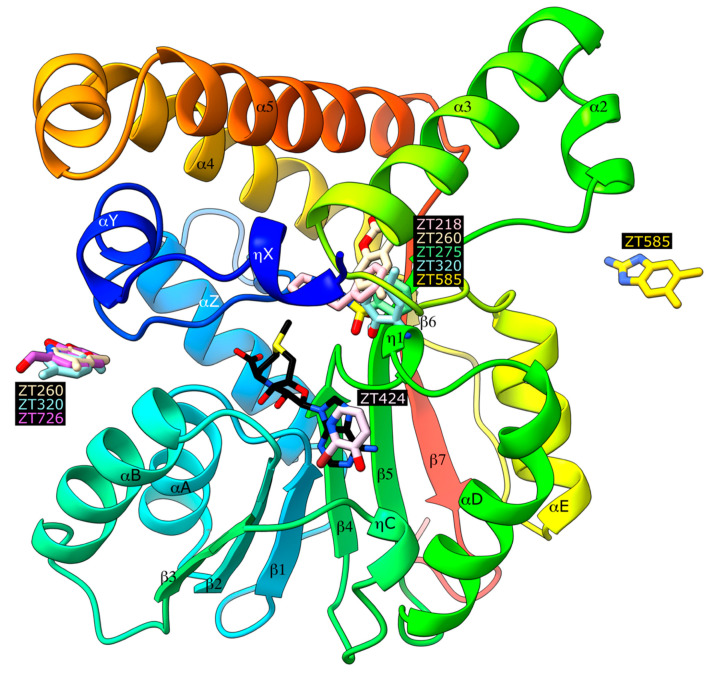
Ribbon representation of the Hma protein (PDB ID 2FK8) in the presence of the S-adenosylmethionine cofactor, represented as sticks with black carbon atoms, with all observed bound fragments represented as sticks with coloured carbon atoms. The protein is represented as a ribbon coloured from blue at the N-terminus to red at the C-terminus and secondary structure elements are labelled according to [30].

Soaking experiments at 20 mM were performed with 126 fragments (average molecular weight 153 ± 29 Da, 0–3 hydrogen bond donors, 0–5 hydrogen bond acceptors, 1–3 cycles, and 0–4 rotatable bonds), providing as many crystals that were flash cooled in a stream of nitrogen gas at 100 K. Diffraction data could be collected for 109 crystals, resulting in 66 datasets with resolution better than 2.5 Å. After a preliminary refinement with dimple, the PanDDA procedure [31,32] identified seven datasets corresponding to possible bound fragments (Figure 1), which were further refined (Table 1). In the case of compound ZT260, as low ligand occupation was observed, a second soaking experiment was performed with 100 mM of compound. Four distinct binding sites were observed (Figure 2): two binding sites are buried in a profound crevice, which has been shown to accommodate the cofactor [30] and the substrate [26], and the other two are on the surface of the protein, involving in one case residues of a neighbouring protein in the crystal.

#### 2.1.1. Fragments ZT218, ZT260, ZT275, ZT320, and ZT585 Bind at the Substrate Binding Site

Five fragments were found to bind Hma at the substrate binding site (Figure 2), where the lipophilic moiety of S-adenosyl-N-decyl-aminoethyl (SADAE) has been observed in Hma [26], as well as didecyldimethylammonium bromide (DDDMAB) and cetyltrimethylammonium bromide (CTAB) in the structures of homologous CmaA1 and CmaA2, respectively [33].

**Figure 3:**
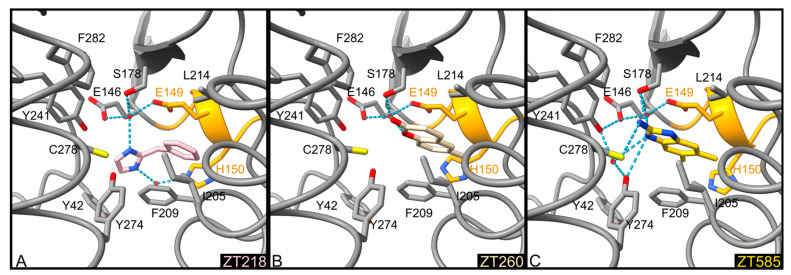
Detailed representation of binding of ZT218 (**A**, pink), ZT260 (**B**, beige), and ZT585 (**C**, yellow) at the substrate binding site. The protein backbone is represented as a tube, the side chains of residues involved in ligand binding are shown as sticks and labelled, water molecules as red spheres, and hydrogen bonds as blue dotted lines. Residues 148–151 forming helix η1 are coloured orange.

**Figure 4:**
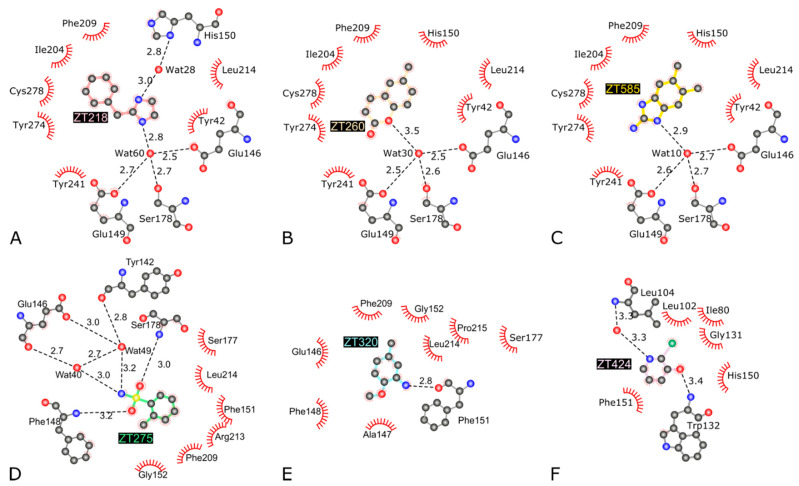
2D interaction maps of fragments ZT218 (**A**), ZT260 (**B**), ZT585 (**C**), ZT275 (**D**), ZT320 (**E**), and ZT424 (**F**) bound to the Hma protein. Hydrogen bonds are represented with dashed black lines and their lengths are indicated. Residues/atoms involved in van der Waals contacts are represented by notched semicircles (figure adapted from LigPlot+ [34]).

The binding modes of ZT218, ZT260, and ZT585 (Figure 1) share common features: these fragments are buried between residues Ile204, Phe209, Tyr274, and Cys278 on one side and residues Glu149, Ser178, and Leu214 on the other side (Figure 3 and Figure 4). Water-mediated hydrogen bonds are observed in all three structures, albeit at longer distance in the case of ZT260: a water molecule, occupying an almost identical position in all three structures, connects the fragments to Glu146OE2 (2.5–2.7 Å), Glu149OE2 (2.5–2.7 Å), and Ser178OG (2.6–2.7 Å). In the case of ZT218, an additional water-mediated hydrogen bond to the imidazole group of His150 is found (Figure 3 and Figure 4). Protein residues interacting with these fragments display a conformation almost identical to that observed in the structures of Hma in the presence of the SAM cofactor or analogues. In the *apo*-Hma structure, residues 151–153 were found to be disordered, resulting in a dramatically different conformation for residues 147–150: the phenyl group of Phe148 in *apo*-Hma is approximately 12.5 Å from the position it occupies in the structures of these complexes. The conformation of residues 147–150 observed in the *apo*-Hma structure would not be compatible with the binding of these fragments. It is likely that the binding of ZT218, ZT260, and ZT585 fragments leads to structuration of residues 147–153, resulting in folding of the helix η1, as already observed upon binding of the SAM cofactor or analogues [26,30].

**Figure 5:**
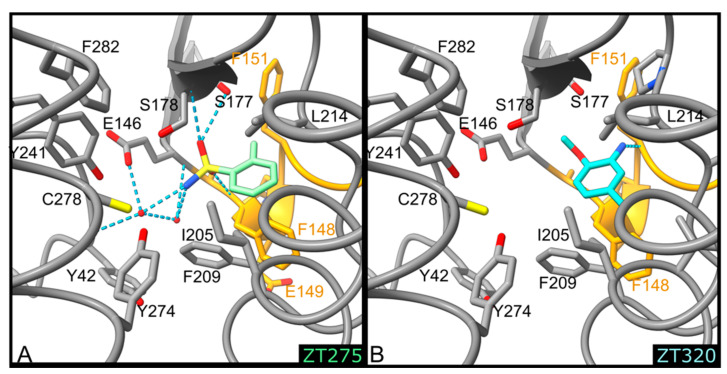
Detailed representation of binding of ZT275 ((**A**), pale green) and ZT320 ((**B**), turquoise) at the substrate binding site. The protein is represented as a ribbon, the side chains of residues involved in ligand biding are shown as sticks and labelled, water molecules as red spheres, and hydrogen bonds as blue dotted lines. Residues 148–151 forming helix η1 are coloured orange.

Although ZT275 and ZT320 (Figure 1) also bind to Hma at the substrate binding site (Figure 2), they induce a previously unobserved conformation for residues 147–154. These two fragments establish van der Waals contacts with Phe148, Gly152, Phe209, and Leu214 (Figure 4 and Figure 5). The oxygen atoms of the sulphonamide group of ZT275 form a hydrogen bond with the main-chain nitrogen atom of Phe148 (3.2 Å) and Ser178 (3.0 Å). In the case of ZT320, the nitrogen atom of the amine moiety forms a hydrogen bond with the oxygen atom of the main chain of Phe151 (2.8 Å, Figure 4 and Figure 5). In both cases, binding results in a modified conformation for residues 147–154. The three-residue long helix η1 (Phe148–His150), observed in the presence of the SAM cofactor or the ZT218, ZT260, or ZT585 fragments, is pushed away from the fragment binding site, reorganises, and includes Phe151. In this new position, Phe148 is about 10 Å away from the position it occupies in the structure of the other complexes: the main-chain atoms are in a position similar to that observed in the *apo*-Hma structure, but the position of the side chain is different, as a result of a 110° rotation of the χ1 dihedral angle. In addition, Glu149 and His150 are found at the position where the adenine moiety of the cofactor resides when bound to Hma [26,30]. Therefore, binding of ZT275 or ZT320 induces a new conformation that would not be compatible with the presence of the cofactor.

#### 2.1.2. Fragment ZT424 Binds at the Cofactor Adenine Site

**Figure 6:**
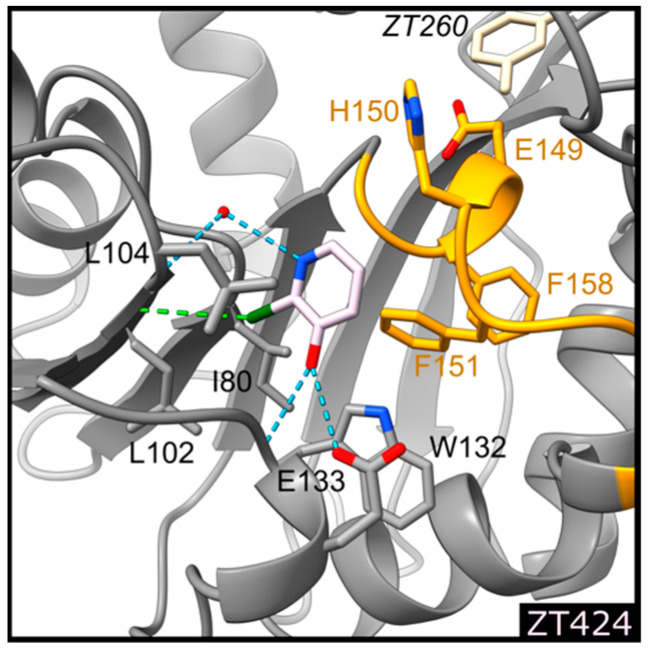
Detailed representation of binding of ZT424 (light pink) at the cofactor binding site. The protein is represented as a ribbon, the side chains of residues involved in ligand biding are shown as sticks and labelled, water molecules as red spheres, and hydrogen and halogen bonds as blue and green dotted lines, respectively. Residues 148–151 forming helix η1 are coloured orange. The position of ZT260, coloured beige, in the substrate binding site is also indicated for easier comparison with Figure 4 and Figure 5.

Binding of ZT424 (Figure 1) is observed at the position where the adenine moiety of the SAM cofactor and its analogues were located [26,30] (Figure 2). ZT424 establishes van der Waals contacts with the side chains of Leu104, Trp132, His150, and Phe151 (Figure 4 and Figure 6). The bromine atom of ZT424 makes a weak halogen bond [35] with the main-chain oxygen atom of Leu102 (4.0 Å), while the ring nitrogen atom of the fragment interact with a water molecule (3.3 Å), which is also hydrogen bonded to the main-chain nitrogen atom of Leu104 (3.3 Å). The hydroxyl group of ZT424 makes a weak hydrogen bond with the main-chain nitrogen atom of Trp132 (3.4 Å) and with the carboxylate group of Glu133 (3.4 Å). In this structure, residues 147–154 display the same conformation as that found in the structures of Hma in the presence of the SAM cofactor or analogues, as well as those obtained in the presence of ZT218, ZT260, and ZT585.

#### 2.1.3. Fragments ZT260, ZT320, ZT585, and ZT726 Bind at the Protein Surface

**Figure 7:**
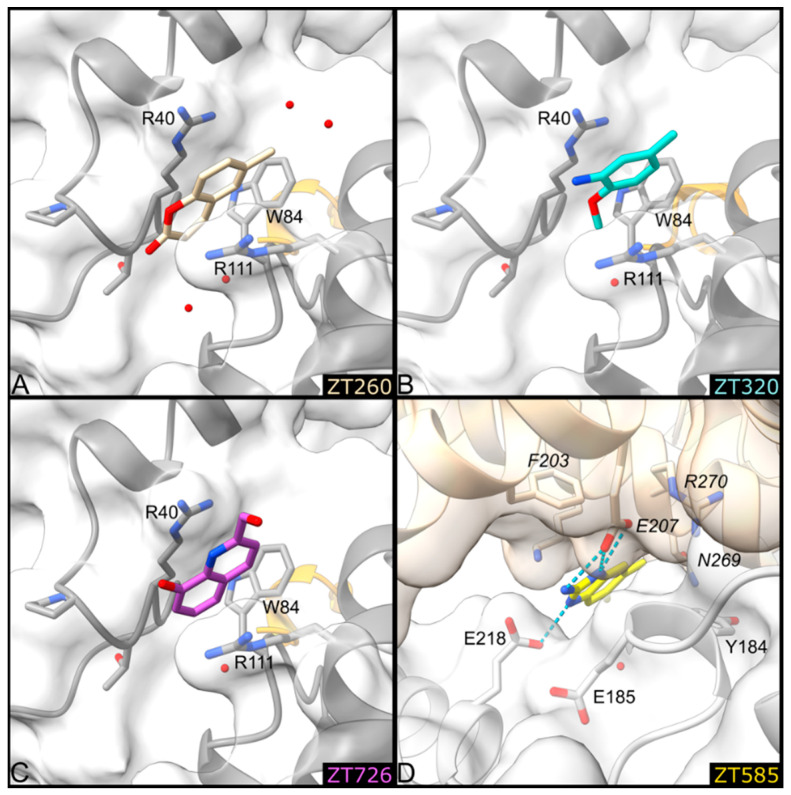
Detailed representation of binding of ZT260 (**A**), ZT320 (**B**), ZT726 (**C**), and ZT585 (**D**) at the protein surface. The protein is represented as a ribbon, the side chains of residues involved in ligand biding are shown as sticks and labelled, water molecules as red spheres, and hydrogen bonds as blue dotted lines. Residues 148–151 forming helix η1 are coloured orange. In D, the symmetry-related molecule is represented in beige and labelled in italics.

Two fragment binding sites are observed on the surface of Hma (Figure 2). The first is delineated by Arg40, Arg111, and Trp84. A second molecule of the ZT260 and ZT320 fragments is located at this position, as well as ZT726 (Figure 1). The planar aromatic ring of ZT260, ZT320, and ZT726 is intercalated between the guanidinium groups of the two arginine residues and forms a perpendicular aromatic–aromatic interaction with the indole moiety of Trp84 (Figure 7). Broad, planar, and ill-defined electron density peaks were observed at this position in several of the structures obtained in this study, but they did not allow the unambiguous positioning of the corresponding fragments.

A second surface binding site is also observed in the case of ZT585, located between two protein molecules in the crystal, near helix α2 (Figure 7). ZT585 makes van der Waals contacts with the side chain of Tyr184 and hydrogen bonding with the side chain of Glu218. ZT585 is also hydrogen-bonded to the side chain of Glu207 and within van der Waals distance of the main-chain atoms of Asn269 and Arg270, all belonging to a symmetry-related molecule in the crystal. The binding of ZT585 induces significant conformational changes. Indeed, in all Hma structures determined so far, the α2 helix comprises residues 183–188 and a two-residue long loop (residues 189–190) connects the α2 and α3 (residues 191–208) helices. In the presence of ZT585, the α2 helix is shortened at residues 183–185 and the α3 helix is extended with an additional turn at its N-terminus (188–208).

### 2.2. Chimeric Compounds

**Figure 8:**
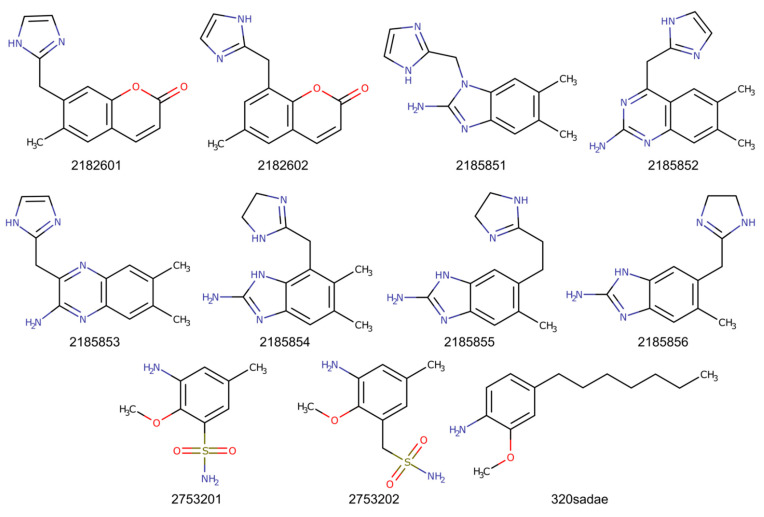
Chimeric compounds derived from merged fragments.

As several fragments bind to the substrate binding site, chimeric compounds were designed to mimic the simultaneous binding of two fragments and thus improve binding affinities. Superimposition of fragments suggested that chimeric compounds could be derived from the fusion of ZT218 with ZT260 or ZT585. Indeed, replacement of the phenyl ring of ZT218 with the aromatic core of ZT260 or ZT585 results in the series 218260x and 218585x (Figure 8). On the other hand, ZT275 could be merged with ZT320 to give the compounds 275320x (Figure 8). Additionally, compound 320sadae was designed by the addition of a lipophilic C_7_ chain to the aromatic nucleus of ZT320, in order to mimic the binding of SADAE [26]. The chimeric compounds were manually positioned in the appropriate structure of Hma using Coot.

### 2.3. Molecular Dynamics Simulations

#### 2.3.1. *Apo*-Hma

In the crystallographic structure of *apo*-Hma, residues 151–153 were found to be disordered [30] suggesting that the 146–155 loop, connecting strand β4 to helix αD, was mobile, at least partially. In the presence of the SAM cofactor [30] and analogues [26], this loop displays decreased mobility, and residues 148–150 form the short η1 helix. A similar conformation of the η1 helix was also observed in the structures of complexes of Hma with ZT218, ZT260, and ZT585 bound in the substrate binding site and with ZT424 bound in the cofactor binding site. On the other hand, a very different conformation was observed in the presence of ZT275 and ZT320 in the substrate binding site. This new conformation would not be compatible with cofactor binding, as Glu149 and His150 are displaced to the position occupied by the adenine moiety. Molecular dynamics simulations were performed for *apo*-Hma, using the two observed conformations of the loop, for a simulation time of 1.2 μs. The calculations were performed in triplicate. The all-atoms root-mean-square fluctuation (RMSF) per residue monitored throughout the simulation indicates that some parts of the protein are indeed more flexible (Figure S1). In both cases, four regions of higher mobility can be identified. Indeed, the N- and C-terminal extremities (residues 19–28 and 298–301, respectively) and residues 152–155 and 182–195 display an average RMSF greater than 1.5 Å for more than three consecutive residues. Residues 152–155 are part of the disordered loop observed in the *apo*-Hma structure that folds upon cofactor binding and residues 182–195 are part of helices α2 and α3. These residues are displaced upon binding of ZT585 at the protein surface (see above). Although the RMSF profiles are comparable for the two starting conformations, residues 129–136 display greater mobility in the conformation compatible with the presence of the cofactor (average RMSF of 1.5 Å compared to 0.9 Å).

The root-mean-square deviation (RMSD) from the starting conformation was also analysed, after removing the global rotational and translational displacement and ignoring the parts of the protein with the highest RMSF, i.e., residues 19–28, 152–155, 182–195, and 298–301. The variations of the RMSD in function of the simulation time (Figure S2) display a homogeneous behaviour independent of the starting conformation. The RMSD converges to about 1.2–1.7 Å after 0.2 μs in each case.

The possibility of a transition between the two conformations observed for residues 147–154 was also investigated. To this end, some distances were monitored during the simulation. This was the case for the distance between the centre of mass of the aromatic side chains of Phe148 and Phe160, which was measured to be 5.7 and 16.2 Å in the crystallographic structures of the cofactor-compatible and cofactor-incompatible conformation, respectively. Similarly, the distances from the centre of mass of the imidazole group of His150 to the Cα atom of Gly131 or to the centre of mass of the aromatic ring of Tyr42 was also monitored. Initial values were 11.5 Å and 9.3 Å, respectively, in the cofactor-compatible conformation, and 4.7 Å and 14.4 Å, respectively, in the cofactor-incompatible conformation. Representative profiles of the variation of these distances over the course of the simulation are shown in Figure S3.

#### 2.3.2. Hma in the Presence of Fragments or of Chimeric Compounds

Molecular dynamics simulations were also performed with Hma in the presence of fragments, starting from observed crystallographic structures, or chimeric compounds, starting from manually generated structures. In the case of the fragments ZT218, ZT260, and ZT585, for which water molecules mediate hydrogen bonds to the protein, two simulations were performed, with or without these water molecules. Simulations were also performed in the presence of the cofactor or analogues of the cofactor, starting from the coordinates of the complexes found at the PDB [26,30].

### 2.4. Estimation of Binding Energies for Fragments and Chimeric Compounds

For the estimation of binding energies, 2000 consecutive frames were selected from the trajectories of the molecular dynamics simulation, after an equilibrium was reached, visualised by the stabilisation of the main-chain RMSD. One frame out of two was used for the calculation of the binding energies, according to the generalised Born method and the Poisson–Boltzmann method coupled to the surface area continuum solvation method (hereafter GBSA and PBSA, respectively). Both approaches approximate the enthalpic term of the binding Gibbs energy and neglect the entropic term, which would be complicated and time consuming to evaluate. Nevertheless, this simplification allows compounds to be ranked in a drug design perspective and the impact of chemical modifications of the ligand on binding to be assessed [36,37]. The PBSA approach is generally considered more accurate in calculating absolute free energies, but it is more time consuming and appears to be more dependent on the system under study, whereas the GBSA approach is better at ranking binding affinities [37]. PBSA and GBSA terms were evaluated for all ligands investigated, including the SAM cofactor and its analogues SAH, SADAE, and sinefungin, which have been shown to bind Hma [26,30]. The binding energies evaluated using the PBSA method are consistently lower than those obtained with the GBSA method (Table 2 and Figure S4). However, as a good correlation was found between the two methods (R^2^ = 0.983, Figure S4), the values obtained with the GBSA method will be considered.

As expected, the estimated binding energies for the fragments are significantly higher (−17.9 to −26.8 kcal/mol) than the values obtained for the SAM cofactor and its analogues (−39.6 to −67.7 kcal/mol). SAM, SAH (the reaction product), and sinefungin display comparable values (−45.7, −40.2, and −39.6 kcal/mol, respectively) while SADAE shows an extremely favourable binding energy (−67.7 kcal/mol). The chimeric compounds resulting from the fusion of the fragments exhibit binding energies ranging from −18.9 to −35.7 kcal/mol, intermediate between values found with the original fragments or the cofactor and its analogues. The most favourable chimeric compounds are 320sadae (−35.7 kcal/mol), which combines ZT320 with a C_7_ alkyl chain reminiscent of the C_10_ alkyl chain of SADAE, and 218585x (−32.3 to −35.1 kcal/mol) resulting from the fusion of ZT218 and ZT585. The chimeric compounds 218260x and 218585x show more favourable binding energies (between −29.9 and –35.1 kcal/mol) than the individual original fragments (−22.8 kcal/mol for ZT218, −18.0 kcal/mol for ZT260, and −26.8 kcal/mol for ZT585), which is not the case for the chimeric compounds 275320x, which exhibit comparable binding energies (between −18.9 and −24.9 kcal/mol) to those found for ZT275 (−17.9 kcal/mol) and ZT320 (−23.0 kcal/mol).

## 3. Discussion

### 3.1. Crystallographic Screening

X-ray crystallography is a powerful technique for fragment screening, as high concentrations of fragments can be achieved in co-crystallisation or soaking experiments, as long as the crystals are resistant to the treatment [38,39,40]. High concentrations are required to provide clear electron density for bound fragments, despite the expected low affinity resulting from their low molecular weights [41,42]. Nevertheless, weak binding is often observed, and specific ligand detection procedures have to be used in order to detect those fragments. In this regard, the use of the PanDDA procedure [31,43] was instrumental in this study to visualise the binding of the ZT275, ZT320, ZT424, and ZT726 fragments that were barely visible in conventional electron density maps.

Crystallographic screening of 126 fragments identified 7 bound fragments, corresponding to a hit rate of 5.5%, in the lower range of what is usually observed in a fragment screening [43,44,45,46]. Among the seven positive hits, five were found to bind in the substrate binding site and one in the cofactor binding site at the adenine position.

### 3.2. Molecular Plasticity of Hma

Comparison of the structure of *apo*-Hma [30] with those of Hma in the presence of the SAM cofactor or analogues [26,30] showed that the 146–155 loop was highly mobile in the absence of ligands and stabilised upon binding of the cofactor or analogues. This is also confirmed by our structures in the presence of the ZT218, ZT260, ZT424, and ZT585 fragments, since the 146–155 loop adopts a similar conformation to that observed in the presence of the cofactor. Surprisingly, while the ZT275 and ZT320 fragments also bind at the substrate binding site, albeit deeper in the crevice, they induce a different conformation of the 146–155 loop. Notably, in this new conformation, Glu149 and His150 occupy the position of the adenine portion of the cofactor. Hence, this new conformation is likely to be incompatible with the presence of the cofactor.

Molecular dynamics simulation of *apo*-Hma, starting with either of the two conformations observed for the 146–155 loop, was run in triplicate for simulation time of 1.2 μs to assess the structural plasticity of each conformation and the possible exchange between them. RMSD analysis along the simulation indicates that both conformations reach an equilibrium state with RMSD values of approximately 1.5 Å relative to the starting conformation (Figure S2). The RMSFs along the protein backbone also display a homogeneous behaviour: in addition to the N- and C-terminal ends, two regions display higher mobility, namely, residues 147–156 and 188–200, as previously observed in the case of the other mycolic acid methyltransferases CmaA2 and CmaA3, which are responsible for the cyclopropanation of MAs [47]. A notable exception occurs for residues 129–137 for which a greater mobility is observed in the case of the cofactor-compatible conformation (average RMSF of 1.5 Å compared to 0.9 Å) (Figure S1). These residues border the cofactor binding site and interact with its adenine moiety [30]. In the cofactor-compatible conformation of *apo*-Hma, this site is filled with water molecules, and residues 129–136 are not restrained. In the cofactor-incompatible conformation, this site is occupied by His150, which makes a water-mediated hydrogen bond with the carboxylate group of Glu133. An additional hydrogen bond is observed between the main-chain oxygen atom of His153 and the side-chain nitrogen atom of Trp132 (2.9 Å). These interactions likely decrease the mobility of residues 129–136. In the presence of the SAM cofactor or analogues, it is the adenine moiety that similarly limits the mobility of residues 129–136 (Figure S1).

The crystallographic structures presented here indicate that the substrate binding site of Hma is capable of adopting at least two distinct conformations, depending on the ligand bound. Interestingly, one of these conformations is not compatible with the presence of the SAM cofactor in its binding site, which, from the perspective of inhibiting the enzyme activity, appears particularly interesting. It seems that there is no transition between the two conformations, at least during the 1.2 μs of the simulation. However, the evolution of inter-residue distances throughout the simulation of *apo*-Hma (Figure S3) suggests that the cofactor-incompatible conformation displays less structural variability than the cofactor-compatible conformation.

### 3.3. Computed Binding Energies of Fragments and Chimeric Compounds

As expected for low molecular weight fragments, the calculated binding energies are rather high (−19.1 kcal/mol on average), suggesting that the interactions are indeed tenuous. The explicit inclusion of experimentally observed water molecules involved in the interactions with the fragment and the protein does not significantly alter the binding energies. Indeed, although the presence of these water molecules might have an effect on the position of the fragment during the dynamics, and thus indirectly affect the estimation of binding energies, they do not directly contribute to the binding energy estimation, as the calculation relies on an implicit solvent model.

Although five of the identified fragments bind to the substrate binding site, they can be divided into two binding modes. Binding of the ZT218, ZT260, or ZT585 fragments induces a conformation for residues 146–155 similar to that observed in the Hma structures obtained in the presence of the SAM cofactor and analogues. Furthermore, from a steric point of view, the binding of these fragments would not prevent cofactor binding. Thus, the inhibitors derived from these fragments would compete with the enzyme substrate. On the other hand, binding of ZT275 and ZT320 fragments induces a different conformation for residues 146–155, resulting in residues 149 and 150 occupying the position where the adenine part of the cofactor is located. Thus, inhibitors derived from these fragments would simultaneously prevent binding of the substrate and cofactor, in a manner similar to that observed for SADAE [26,48]. However, unlike SADAE, which competes for binding with both substrate and cofactor, the inhibitors derived from the ZT275 and ZT320 fragments would act as competitive inhibitors for the substrate but as allosteric inhibitors for the cofactor.

Based on the observed structures, several chimeric compounds were designed by fusion of the identified bound fragments. The 218260x and 218585x series were derived from merging fragments ZT218 with ZT260, and ZT218 with ZT585, respectively, and compounds 275320x resulted from merging ZT275 and ZT320. Binding energies of chimeric compounds were evaluated in the same way as for those of original fragments. Among those chimeric compounds, compounds 2753201 and 2753202 display binding energies of the same order of magnitude as those of the original fragments (−18.9 and −24.9 kcal/mol). This could be related to the low molecular complexity of these compounds, comparable to that of the original fragments. The chimeric compounds 218260x and 218585x display much more favourable binding energies (−31.5 and −33.9 kcal/mol, on average, respectively).

SADAE is a SAM analogue that was shown to inhibit *Escherichia coli* cyclopropane fatty acid synthase (CFAS) both in vivo and in vitro [48], as well as Hma in vitro [26]. Furthermore, SADAE also inhibited the growth of Mtb and *M. smegmatis*, indicating that it is able to cross the cell wall of mycobacteria [26]. The efficacy of SADAE has been attributed partly to the lipophilic C_10_ chain, which is thought to mimic the lipophilic chain of CFAS and Hma substrates [26,48]. The calculated binding energy for SADAE is extremely favourable, due to the numerous van der Waals interactions resulting from the presence of the lipophilic chain. It should be noted, however, that for a compound with such a degree of freedom, neglecting the entropic term is likely to lead to significant approximations. The chimeric compound 320sadae was designed by adding a C_7_-chain to ZT320 to occupy the substrate binding site. It exhibits the lowest binding energy (−35.7 kcal/mol), compared to other chimeric compounds, marginally better than the values obtained for compounds of the 218585x series. Compared to the binding energy of the original ZT320 fragment (−23.0 kcal/mol), the observed gain is, however, important, even considering the uncertainty resulting from neglecting the entropic term.

These results suggest that at least two strategies are conceivable to inhibit Hma, and potentially other mycolic acid methyltransferases, which share high structural similarities [33,49]. First, elaborating from the ZT275 and ZT320 fragments would yield compounds that simultaneously interfere with substrate binding, as they occupy the substrate binding site, and prevent cofactor binding, as they induce structural modifications of the protein that are not compatible with the presence of SAM. Secondly, the addition of a lipophilic moiety would optimise the occupation of the substrate binding site, and would contribute to improve the specificity of the compounds towards methyltransferases acting on long aliphatic compound, such as lipids. In this regard, the functionalisation of the aliphatic chain that would mimic reaction intermediates would further improve the inhibitors’ affinity and specificity.

## 4. Materials and Methods

### 4.1. Expression, Purification, and Crystallisation of Hma

The Hma protein was expressed and purified as previously described [26,48]. In summary, a pET15b plasmid (Novagen) containing the *hma* cDNA was used for transformation of *Escherichia coli* BL21(DE3)pLysS bacteria. This construct exchanges the first three residues of Hma with a 20-residue cleavable His-tag. Expression of the recombinant protein was induced by the addition of 1 mM Isopropyl β-D-1-thiogalactopyranoside (IPTG) at 310 K for 3 h. After sonication and centrifugation, the soluble fraction was loaded onto a nickel affinity column (Amersham Biosciences, Amersham, UK) and the His-tagged protein was eluted with a 5–500 mM imidazole gradient in a buffer consisting of 50 mM MES, pH 6.5, and 300 mM NaCl. A final size exclusion chromatography step, using a Sephadex 75 HiLoad column (Amersham Biosciences), yielded a pure protein for structural studies.

The purified Hma protein was crystallised at 285 K by vapor diffusion using the hanging drop technique. The crystallisation conditions were optimised from those published previously [26,30] to reproducibly provide sufficient quantities of good quality crystals. A 3 μL droplet was prepared by mixing 2 μL of a 3–4 mg/mL of Hma solution (MES 50 mM, NaCl 50 mM, pH 6.5) with 1 μL of reservoir solution (BisTris 50 mM, PEG 3350 4% (*w*/*v*), pH 6.5). Under these conditions, seeding of crushed crystal fragments was necessary because the protein concentration in the drop was not sufficient to allow spontaneous nucleation. This procedure yielded reproducibly 5 to 10 single crystals per drop, bipyramidal in shape, and about 200 μm long in their largest dimension, suitable for soaking experiments.

### 4.2. Fragments

A 352-fragment library was acquired from Zenobia Therapeutics. The molecular fragments (average molecular weight 154 ± 29 Da, 0–3 hydrogen bond donors, 0–6 hydrogen bond acceptors, 0–3 cycles, and 0–5 rotatable bonds) were formulated at 200 mM in pure DMSO. Fragments were used without prior purification or characterisation.

### 4.3. Crystallographic Screening

Crystallographic screening was performed by soaking Hma crystals overnight in 20 mM fragment solutions in 50 mM BisTris, PEG 3350 4% (*w*/*v*), pH 6.5, at 285 K. Soaked crystals were cryoprotected by immersion for 2 min in the crystallisation solution supplemented with 20% (*v*/*v*) glycerol before cooling in a stream of nitrogen gas at 100 K.

Diffraction data were collected at ALBA (Barcelona, Spain, beamline XALOC), SOLEIL (Saclay, France, beamline PX1), and European Synchrotron Radiation Facility (ESRF, Grenoble, France, beamlines ID14-1, ID14-2, ID23-1, ID23-2, and ID29), and processed with XDS [50] and AutoProc [51]. Preliminary refinement was performed using the dimple pipeline of the CCP4 Program Suite [52] starting with *apo*-Hma coordinates [30] before identifying structures with potentially bound fragments with the PanDDA procedure [31]. These structures were further refined with REFMAC5 [53], Buster [54], and Coot [55]. The fragment dictionaries were generated using MarvinSketch [56] and the Grade Server [57].

### 4.4. Molecular Dynamics Simulation

Available crystallographic structures of the apo protein and of complexes [26,30], including those described here, as well as models of complexes generated in the presence of the chimeric compounds, were used as starting point for molecular dynamics simulations. The chimeric compounds were drawn and converted in 3D with MarvinSketch [56].

The tleap module for AMBER-20 [58] was used to generate a periodic cubic box extending 10 Å around the protein, containing the structure of the protein, the ligand if present, water molecules represented with the TIP3P model, and sodium cations to neutralise the system. The GPU version of the PMEMD module available in AMBER-20 was used for energy minimisation and molecular dynamics calculations. An initial energy minimisation was performed, with progressively reduced constraints on protein atom positions, followed by a 150 ps equilibration MD and a 100 ns production run. In the case of *apo*-Hma and of the SAM-Hma complex, the production simulations were extended to 1 μs. Analysis of trajectories, as well as monitoring of interactions and of inter-residue distances along the trajectories were performed using CCPTRAJ [59].

### 4.5. Relative Binding Affinity Evaluation

Molecular dynamics (MD) simulation was coupled with the MM-GB/PBSA post-processing method [60] to estimate the interaction energies of fragments, cofactor and cofactor analogues, and chimeric compounds derived from the identified bound fragments. This procedure relies on frames extracted from an all-atom molecular dynamics simulation of a protein–ligand complex, after removal of solvent molecules, since these methods rely on an implicit solvent model. The enthalpic term of the Gibbs free energy of binding is approximated from the force-field energy, and the entropic term is usually neglected as it is extremely time consuming to calculate [61]. Therefore, this procedure does not provide true binding energies, but it can still estimate relative binding energies between ligands, as the entropy term should be dominated by the protein contribution, which should be comparable for the different ligands. The MMPBSA.py.MPI program [60] was used for the calculations.

## 5. Conclusions

The crystallographic screening of a fragment library allowed for the identification of 7 fragments bound to Hma. The presence of bound fragments in the substrate binding site of Hma induced two distinct conformations of residues 147–154. One of these conformations would be incompatible with the presence of the SAM cofactor in its binding site. Second generation chemical compounds were designed based on the observed positions of the fragments. Binding energies of initial fragments, of second generations molecules and of the SAM cofactor and analogues were estimated using MM-GBSA/PBSA methods. Whereas bonding energies of fragments were high, as would be expected for low molecular weight compounds, some of the second generations compounds displayed binding energies close to that found for cofactor analogues. These results suggest that our compounds could be further improved to inhibit Hma, and possibly other MAMTs. Additionally, our findings allow to envision the possibility of allosteric inhibition of cofactor binding.

## Figures and Tables

**Table 1:** Data collection and refinement statistics.

	ZT218	ZT260	ZT275	ZT320	ZT424	ZT585	ZT726
PDB code	7Q2B	7Q2C	7Q2H	7Q2D	7Q2E	7Q2F	7Q2G
Data Collection							
Beamline	ESRF, ID14-4	ESRF, ID14-4	ESRF, ID23-1	ESRF, ID29	ESRF, ID29	SOLEIL, PX1	ESRF, ID14-4
Spacegroup	*P*3_1_21	*P*3_1_21	*P*3_1_21	*P*3_1_21	*P*3_1_21	*P*3_1_21	*P*3_1_21
Unit cell a, c (Å)	57.29, 206.00	57.11, 205.90	56.62, 207.67	57.02, 207.35	55.77, 207.02	57.11, 204.46	57.06, 205.93
Resolution range (Å) ^1^	40.21–1.85 (1.96–1.85)	35.66–1.85 (1.96–1.85)	49.03–1.75 (1.86–1.75)	49.38–1.90 (2.02–1.90)	49.17–2.00 (2.12–2.00)	49.46–1.85 (1.88–1.85)	40.10–2.00 (2.12–2.00)
No. unique reflections	34,449 (5438)	33,875 (5254)	40,075 (6368)	31,853 (5032)	27,163 (4292)	33,526 (1649)	27,352 (4359)
Completeness (%)	99.8 (99.7)	98.4 (96.7)	99.5 (99.7)	100.0 (100.0)	99.6 (99.7)	98.3 (99.9)	99.9 (99.9)
Redundancy	6.0 (6.0)	5.1 (3.7)	8.9 (8.8)	11.4 (11.8)	6.7 (6.5)	5.9 (5.2)	7.5 (7.6)
<*I/σ(I)*>	11.5 (1.8)	15.1 (1.0)	13.7 (2.6)	16.2 (2.6)	17.3 (2.4)	7.2 (1.4)	11.4 (1.3)
*R*_merge_ (%)	8.2 (95.5)	5.1 (109.3)	8.9 (75.8)	8.1 (90.9)	5.2 (70.8)	15.8 (116.7)	9.1 (133.9)
CC(1/2)	99.6 (75.2)	99.9 (59.1)	99.6 (89.0)	99.8 (89.2)	99.9 (84.2)	98.3 (53.1)	99.6 (81.4)
Refinement							
Resolution range (Å)	40.25–1.85	35.66–1.85	49.03–1.75	49.38–1.90	49.17–2.00	49.46–1.85	40.10–2.00
No. reflections (work/test)	29,899/1703	28,298/1619	35,907/2034	28,849/1645	26,984/1545	30,090/1718	20,616/1191
*R*_work_/*R*_free_	0.1668/0.2059	0.1792/0.2256	0.1782/0.2105	0.1856/0.2281	0.1847/0.2375	0.1894/0.2293	0.1926/0.2538
No. of non-hydrogen atoms	2484	2472	2525	2440	2385	2480	2339
Protein	2298	2310	2305	2305	2281	2291	2264
Fragment	12	24	11	20	8	24	13
Solvent	174	138	209	115	96	165	62
Rms deviations							
Bond length (Å)	0.007	0.004	0.003	0.005	0.005	0.004	0.008
Bond angles (°)	1.338	1.233	1.164	1.225	1.296	1.199	1.226
Ramachandran plot							
Most favoured (%)	98	98	98	96	98	98	97
Allowed/Outliers (%)	2/0	2/0	2/0	4/0	2/0	2/0	3/0

^1^ Values in parentheses are for the highest resolution shell.

**Table 2:** Estimated binding energies and standard deviations (kcal/mol) for all ligands mentioned in this study.

Ligand	GBSA	PBSA
ZT218	−22.8 ± 2.2	−11.9 ± 2.6
ZT218 *	−22.2 ± 1.8	−10.2 ± 2.2
ZT260	−18.0 ± 1.8	−12.0 ± 2.1
ZT260 *	−22.2 ± 1.8	−10.2 ± 2.2
ZT275	−17.9 ± 2.1	−7.0 ± 2.1
ZT320	−23.0 ± 2.1	−12.8 ± 2.2
ZT424	−18.9 ± 1.7	−9.7 ± 1.9
ZT585	−26.8 ± 1.7	−14.8 ± 2.0
ZT585 *	−26.2 ± 1.7	−14.0 ± 2.0
ZT726	−19.2 ± 1.8	−11.4 ± 2.1
2182601	−29.9 ± 2.5	−10.7 ± 3.0
2182601 *	−33.9 ± 2.2	−19.6 ± 2.2
2182602	−30.5 ± 1.8	−14.2 ± 2.1
2182602 *	−31.5 ± 2.1	−15.6 ± 2.7
2753201	−24.9 ± 2.9	−12.5 ± 2.2
2753202	−18.9 ± 3.8	−5.3 ± 2.6
2185851	−32.4 ± 2.1	−12.2 ± 2.3
2185852	−32.3 ± 2.6	−13.2 ± 2.8
2185853	−34.4 ± 2.0	−14.5 ± 2.4
2185854	−34.3 ± 2.2	−18.2 ± 2.4
2185855	−35.1 ± 2.2	−18.6 ± 2.6
2185856	−35.0 ± 2.3	−19.7 ± 2.5
320sadae	−35.7 ± 2.2	−20.3 ± 2.5
SAM	−45.7 ± 4.9	−25.0 ± 4.1
SAH	−40.2 ± 3.4	−21.8 ± 3.2
Sinefungin	−39.6 ± 3.8	−24.8 ± 3.6
SADAE	−67.7 ± 3.5	−35.8 ± 3.1

*An asterisk following the name of the fragment indicates that experimentally observed bridging water molecules were conserved in the molecular dynamics simulations.

## Data Availability

The atomic coordinates and crystallographic structure factors of complexes described in this work have been deposited in the Protein Data Bank (www.rcsb.org) with accession codes as follows: Hma-ZT218 7Q2B; Hma-ZT260 7Q2C; Hma-ZT275 7Q2H; Hma-ZT320 7Q2D; Hma-ZT424 7Q2E; Hma-ZT585 7Q2F; and Hma-ZT726 7Q2G.

## Supplementary Materials

The following are available online at https://www.mdpi.com/article/10.3390/ph14121282/s1, Figure S1: Root-mean-square fluctuation along the MD simulation of *apo*-Hma. The per residue main-chain atom fluctuations are average of three independent 1.2 µs simulations of *apo*-Hma starting either from the cofactor-compatible conformation (orange) or from the conformation observed in the presence of ZT275 or ZT320 that would be incompatible with the binding of the cofactor (blue). For comparison, the per residue main-chain atom fluctuations of the structure of Hma in complex with SAM (averaged from 2 independent 1.2 µs simulations) is shown in grey. Secondary structures elements are indicated, labelled and coloured as in Figure 2. Figure S2: Evolution of the root-mean-square deviation along the MD simulation of *apo*-Hma. RMS deviations (Å) were computed using main-chain atoms of residues 29–151, 156–181, and 196–297 on the whole trajectory and plotted as a function of time for the 1.2 µs simulation of *apo*-Hma starting either from the cofactor-compatible conformation (blue) or from the cofactor-incompatible, ZT320-bound conformation (orange). For clarity, only one in 10 values is plotted. A single representative curve is displayed for each simulation performed in triplicate. Figure S3: Variations of selected inter-residues distances along the simulation trajectory of *apo*-Hma. Distances were measured between the centre of mass of the aromatic side chain of Phe160 and of Phe148 (blue) and between the centre of mass of the imidazole group of His150 and either the Cα atom of Gly131 (orange) or the centre of mass of the aromatic ring of Tyr42 (grey). Distances are plotted as a function of time for the simulation starting from the cofactor-compatible conformation (top) and from the cofactor-incompatible, ZT320-bound conformation (bottom). Simulations were performed in triplicate, curves from a single simulation are shown. Figure S4: Comparison of binding affinities as evaluated using the GBSA or the PBSA approach. The linear fit is indicated. Initial fragments are represented with red dots, chimeric compounds with blue dots and SAM and analogues with green dots. An asterisk following the name of the fragment indicates that experimentally observed bridging water molecules were conserved in the molecular dynamics simulations. Abbreviation: SIN, sinefungin. A straight line is fitted to all the points, passing at the origin. The square of the Pearson correlation coefficient is indicated.
