# Supplementary material for: Fragment-Based Ligand Discovery Applied to the Mycolic Acid Methyltransferase Hma (MmaA4) from Mycobacterium tuberculosis: A Crystallographic and Molecular Modelling Study
Source: Pharmaceuticals (Basel). 2021 Dec 8;14(12):1282. doi: 10.3390/ph14121282 (PMC8708032; doi:10.3390/ph14121282)
Supplement: Supplementary file 1 [file pharmaceuticals-14-01282-s001.zip › pharmaceuticals-1478733-supplementary.pdf]

Fragment-Based Ligand Discovery Applied to the Mycolic Acid Methyltransferase Hma (MmaA4) from *Mycobacterium tuberculosis*: A Crystallographic and Molecular Modelling Study

Romain Galy [1], Stéphanie Ballereau [2], Yves Génisson [2], Lionel Mourey [1], Jean-Christophe Plaquevent [2] and Laurent Maveyraud [1,*]

[1] Institut de Pharmacologie et de Biologie Structurale, Université Toulouse III - Paul Sabatier, Centre National de la Recherche Scientifique, 31077 Toulouse, France; lionel.mourey@ipbs.fr (L.Mo.)

[2] Laboratoire de Synthèse et Physico-Chimie de Molécules d'Intérêt Biologique, Université Toulouse III - Paul Sabatier, Centre National de la Recherche Scientifique, 31062 Toulouse, France; ballereau@chimie.ups-tlse.fr (S.B.); genisson@chimie.ups-tlse.fr (Y.G.)

* Correspondence: laurent.maveyraud@ipbs.fr; Tel.: +33-561-17-54-35

Supplementary Materials

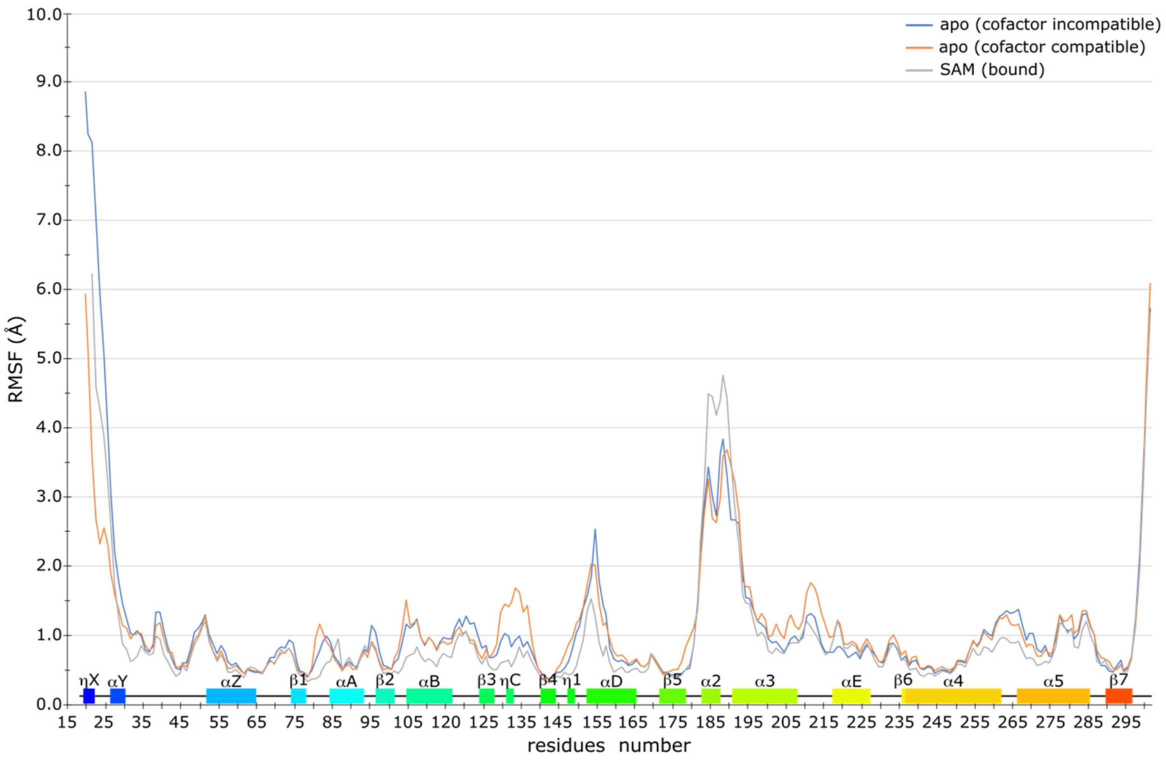

Figure S1. Root-mean-square fluctuation along the MD simulation of *apo*-Hma. The per residue main-chain atom fluctuations are average of three independent 1.2 µs simulations of apo-Hma starting either from the cofactor-compatible conformation (orange) or from the conformation observed in the presence of ZT275 or ZT320 that would be incompatible with the binding of the cofactor (blue). For comparison, the per residue main-chain atom fluctuations of the structure of Hma in complex with SAM (averaged from 2 independent 1.2 µs simulations) is shown in grey. Secondary structures elements are indicated, labelled and coloured as in Figure 2.

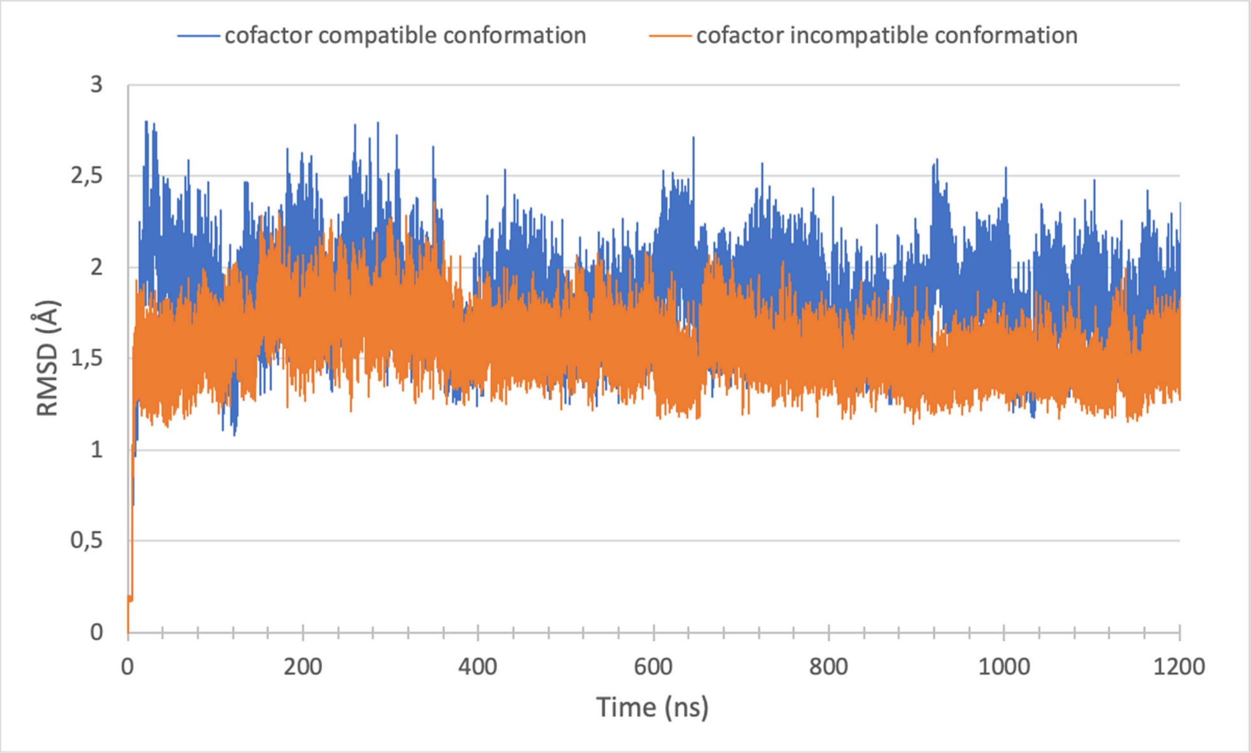

Figure S2. Evolution of the root-mean-square deviation along the MD simulation of *apo*-Hma. RMS deviations (Å) were computed using main-chain atoms of residues 29–151, 156–181, and 196–297 on the whole trajectory and plotted as a function of time for the 1.2 µs simulation of *apo*-Hma starting either from the cofactor-compatible conformation (blue) or from the cofactor-incompatible, ZT320-bound conformation (orange). For clarity, only one in 10 values is plotted. A single representative curve is displayed for each simulation performed in triplicate.

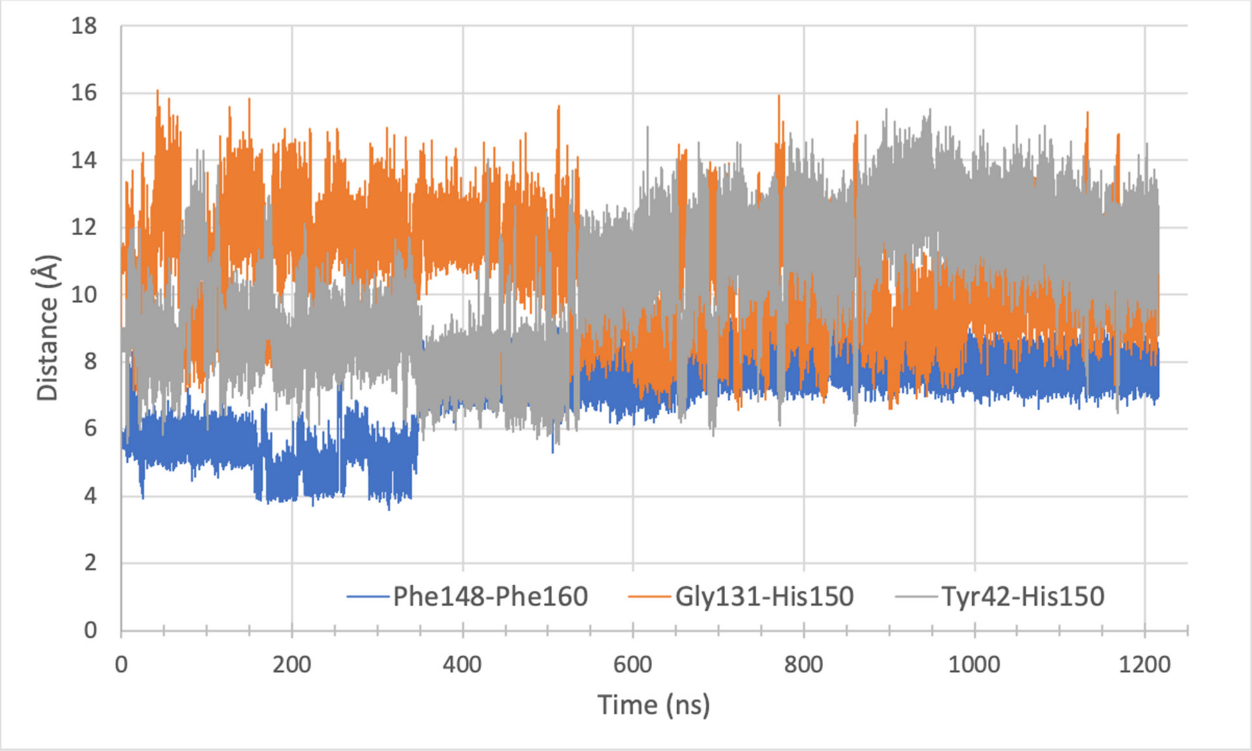

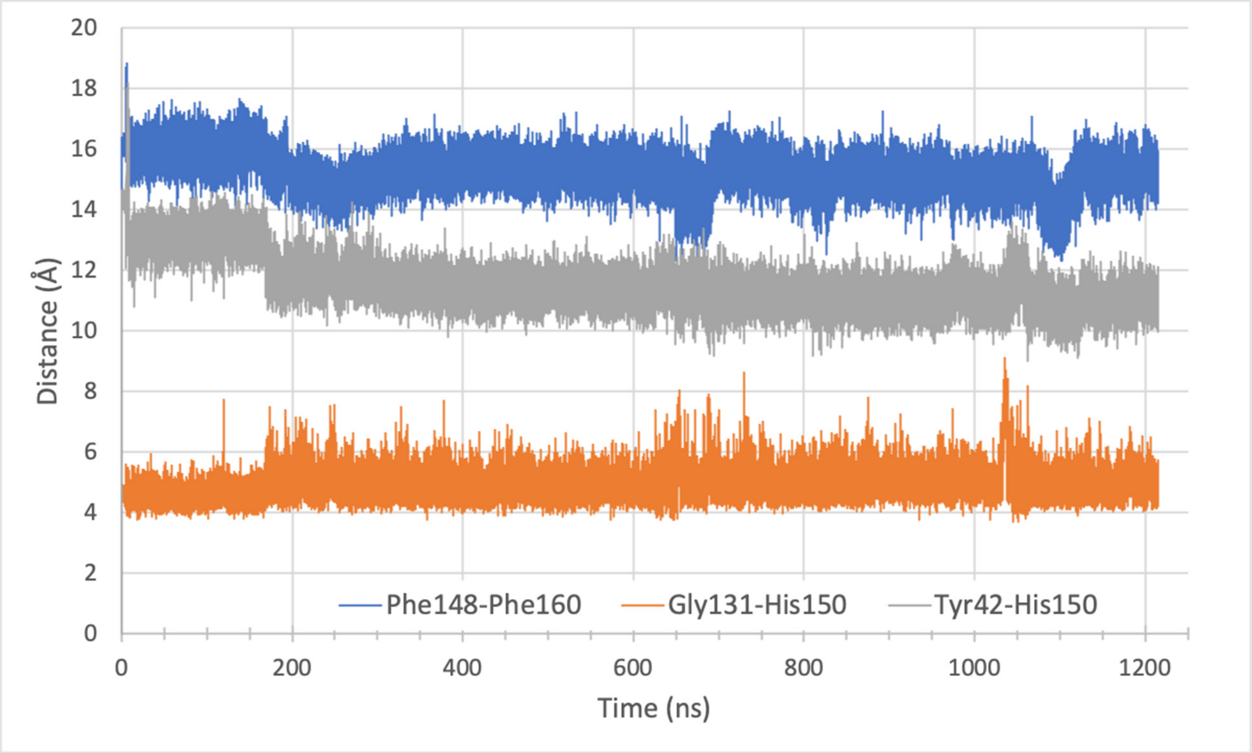

Figure S3. Variations of selected inter-residues distances along the simulation trajectory of *apo*-Hma. Distances were measured between the centre of mass of the aromatic side chain of Phe160 and of Phe148 (blue) and between the centre of mass of the imidazole group of His150 and either the Cα atom of Gly131 (orange) or the centre of mass of the aromatic ring of Tyr42 (grey). Distances are plotted as a function of time for the simulation starting from the cofactor-compatible conformation (top) and from the cofactor-incompatible, ZT320-bound conformation (bottom). Simulations were performed in triplicate, curves from a single simulation are shown.

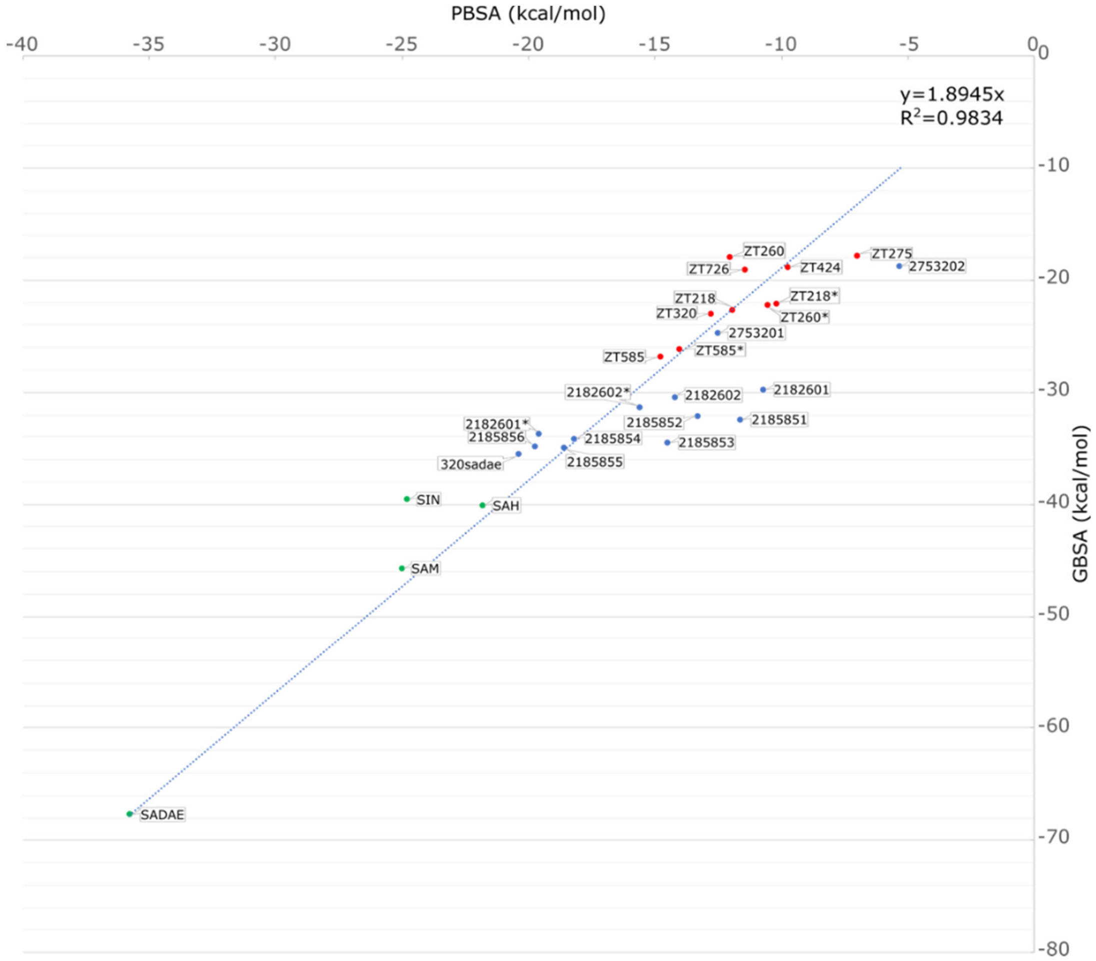

Figure S4. Comparison of binding affinities as evaluated using the GBSA or the PBSA approach. The linear fit is indicated. Initial fragments are represented with red dots, chimeric compounds with blue dots and SAM and analogues with green dots. An asterisk following the name of the fragment indicates that experimentally observed bridging water molecules were conserved in the molecular dynamics simulations. Abbreviation: SIN, sinefungin. A straight line is fitted to all the points, passing at the origin. The square of the Pearson correlation coefficient is indicated.
